# Supplementary material for: Chromothripsis during telomere crisis is independent of NHEJ, and consistent with a replicative origin
Source: Genome Res. 2019 May;29(5):737–49. doi: 10.1101/gr.240705.118 (PMC6499312; doi:10.1101/gr.240705.118)
Supplement: Supplemental Material [file supp_gr.240705.118_Supplemental_file_1.zip › contigs/annotated_contigs/DB68/contig.3.DB68_length_665_mean_cov_10.962406015.docx]

**DB68_length_665_mean_cov_10.962406015**

```
TTATAAGGTACCTGAGATACTTTGATACAGGCATATAATATGTAATCATTATGTGATAGAAAATGTGGTATCTATCCCCTCCAGCATTT
 >chr7:66603355-66603680 + E=9e-178
ATCCTTTGTGTTACAAACAATCCAATTATACTCCTTTAGTCACTTTTAAATGTACAATTAAATTATGTTTGACTATATTGTCAACCTGT

TGTGCTATCAAAGACTAGGTCTTATTCATTTTTTCTATTTTTTGTACCCATTAACCATCCCACCTTCCTCCCAACACCCTCACTACCCT

TCCCAGCCTCTGGTAACCATCCTTCTACTGTCTATCTCCATGAGTTGAATTGTTTTG|A|ACAACACCTAAAACTTAAATAAATACTGT
                                                          >chr7:66099179-66099343 - E=6
AATACCAGCATTTTAGGAGGCCAAGGTGGGAGGACTGCCTAAGGCCAGGAGCTTGAGACCAGTCTGGGCAACACAGCAAGATCCTATCT
e-87 p=0e+00
TTATTTTTATAGATAGATAGATAGATAG|ATAGATAGATAGATAGA|TAGATAGATAGATAGATAGAATCACACACATCAAGACCAGTG
                             >chr7:66099018-66099216 - E=6e-99
GAAGCCTCAGTGCATCAGAGCTCATATATATACATATATATATATATATATATGTATACACACACCCATATATACATATATATATATGT

GTGCATGTATGTGTACATCTGTGTGTGTGGGTGTATAATGAGATAA
```
